# Editorial: Educating Health Professionals in Genomic Medicine: Evidence-Based Strategies and Approaches

**DOI:** 10.3389/fgene.2020.00696

**Published:** 2020-08-07

**Authors:** Sylvia A. Metcalfe, Michael J. Dougherty, Clara L. Gaff

**Affiliations:** ^1^Murdoch Childrens Research Institute, Royal Children's Hospital, Melbourne, VIC, Australia; ^2^Department of Pediatrics, Melbourne Medical School, University of Melbourne, Melbourne, VIC, Australia; ^3^School of Medicine, University of Colorado, Aurora, CO, United States; ^4^Melbourne Genomics Health Alliance, Parkville, VIC, Australia

**Keywords:** genomics education, evaluation, needs assessment, continuing professional education, program logic

With the rapid advancement of genomic technologies, particularly in the area of testing for human disease, genomics is being increasingly integrated into clinical care across many health disciplines. Nonetheless, there has been perceived lack of relevance by some non-genetic specialist health professionals and many challenges exist for genomic medicine to be successfully implemented (Joyner and Paneth, 2019). All specialists in genomic medicine will play an important role in preparing their non-specialist colleagues for this transformation in clinical care. New and innovative strategies both for education and system change will be required.

This special topic focusses on how evidence-based strategies and approaches can be used to develop and successfully implement education of health professionals in genomic medicine. This issue includes 12 articles on education in African countries, Australia, Canada, England, the Netherlands, Sri Lanka, and the United States of America, covering the educational needs of health care providers in genomic medicine and examples of emerging and successful educational activities, including their development, implementation, evaluation and outcomes.

The issue begins with two mini-review articles. The first, by Crellin et al., discusses the important role that a person's perceived need for learning plays in effective education, which we know from adult learning theory. They therefore reviewed the literature examining medical specialists' perceptions of genomics, drawing on studies from the earlier “genetic” era (due to the paucity of empirical studies published to date in the “genomic” era). They emphasize that the educational needs of medical specialists should be investigated to determine “if there is a need” and “how to meet the need,” before tailoring educational interventions, and to encourage that education be considered part of a wider clinical implementation strategy. In her mini-review, Cornel et al. summarizes more than 30 years of research in Amsterdam on education for non-genetics experts. She notes that while some improvements have been seen in genetic competence, subsequent impacts on clinical practice and population health have been challenging to measure.

Nisselle et al. expand on the challenges of measuring the effectiveness of genomics education and advocate the use of program logic to develop and evaluate education interventions. Program logic models can help describe the inputs (such as stakeholder engagement and needs assessments), activities (such as development and delivery of the program), and intended outcomes of a program. Program logic models also include where and how evaluation can be targeted. The authors describe the development of a generic program logic for genomics education that took place at a workshop with international experts in 2018 in Melbourne, Australia. They then report the results of testing the program logic in four diverse educational contexts and show that the model can be applied as a tool in multiple ways.

Several articles report on needs assessments to inform targeted education to a variety of health professionals. Saleh et al. conducted qualitative interviews with nurses, midwives, and allied health professionals in Australia to identify perceived genetic knowledge and education needs. They found that there was interest in genomics, tempered with uncertainty around how to access reliable resources and how to deal with challenges in incorporating education in clinical practice. In a separate large qualitative needs assessment with medical specialists in Australia, McClaren, Crellin et al. found that their participants believed confidence and skills in genomics clinical care require experiential learning (i.e., learning through reflection on doing); this mode of learning also includes interacting with their peers, especially “genomic champions,” experts in their own specialty who have gained genomics expertise. Further findings from this study informed the development of a national survey, which is described in a second article by McClaren, King et al.. This paper describes the methodology, which used a mixed-methods approach and included additional interviews with education providers and a Delphi panel of experts, followed by piloting. To add to the rigor of survey development, the items were also informed by a theoretical framework of behavior change, the COM-B model: capability, opportunity, motivation and behavior.

In England, as genomic medicine is being rolled out through the National Health Service, there is a national coordinated approach to educating and upskilling health professionals. To inform these programs, Simpson et al. undertook a cross-professional training needs analysis using a national survey and found that online learning was preferred by many. Their findings are providing an evidence base to inform resource development and an understanding of the motivations to engage in learning, which can aid in resource design. Among the suite of resources produced in England is a 3-week Massive Open Online Course (MOOC) on whole genome sequencing. Bishop et al. describe the rationale for choosing this type of learning resource, the process of development, including the recruitment and training of mentors, and the short-term evaluation outcomes.

Carroll et al. focused on primary care practitioners (family physicians) in Canada. They used a questionnaire to understand current involvement and confidence in genomic medicine, as well as attitudes about clinical validity, how genomic medicine could be integrated into primary care practice, and necessary resources and education. Their findings have informed the development of a website containing evidence-based resources, including point-of-care tools.

Clinical decision making and information for genomics education purposes can also be supported by tools embedded into electronic health records. Williams et al. in the USA describe some of the barriers to the effective use of electronic health records in supporting the clinical practice of genomics, and they identify “lessons learned” and several testable, potential solutions.

The studies and activities discussed so far are based in developed countries. In their opinion piece, Sirisena and Dissanayake from Sri Lanka begin by articulating the challenges of integrating genomic medicine in low- and middle-income countries and then discuss strategies for genomics education in these countries. In the final paper to be mentioned in this editorial, Nembaware et al. report on developments of the African Genomic Medicine Training Initiative (AGMT), in which they report on a program of training for nurses across 11 countries in Africa. They describe undertaking both a general and a targeted needs assessment and the construction of nurse personas to develop and map core competences adapted to the needs of the African continent. These personas and competences then informed the curriculum and evaluation plan. A blended-learning course was subsequently implemented using trained community-based facilitators in virtual and physical classrooms in 19 different sites across Africa, with outcomes to be reported in due course.

Taken collectively, it is evident that major stakeholders in genomic healthcare systems recognize the importance of evaluation in education delivery and outcomes. Several examples include those responsible for health professional education (e.g., Health Education England, the Centre for Genetics Education, Australia), the professional genetics community (e.g., African Genomic Medicine Training Initiative), research networks (e.g., eMERGE), academic researchers and networks (e.g., Australian Genomics Health Alliance), and clinical providers (e.g., those using resources produced by Genetics Education Canada-Knowledge Organization and Geisinger's GenomeFIRST program). The challenges of ensuring sustained, effective education at a scale that ultimately and significantly improves patient care makes robust evaluation all the more important. Education funders, as well as those delivering education, need to be confident that an approach is—or can be—effective. The use of frameworks, such as those proposed by Nisselle et al., could move the field from evaluation of individual education programs to meta-analysis, yielding a robust body of knowledge to guide educators about interventions with strong evidence of effectiveness. This will advance the ultimate goal of improved patient care by educating clinicians about best practices in genomic medicine.

## Author Contributions

All authors listed have made a substantial, direct and intellectual contribution to the work, and approved it for publication.

## Conflict of Interest

The authors declare that the research was conducted in the absence of any commercial or financial relationships that could be construed as a potential conflict of interest.
